# Whole-genome methylation analysis of benign and malignant colorectal tumours

**DOI:** 10.1002/path.4132

**Published:** 2013-01-24

**Authors:** Andrew D Beggs, Angela Jones, Mona El-Bahwary, Muti Abulafi, Shirley V Hodgson, Ian PM Tomlinson

**Affiliations:** 1 Molecular and Population Genetics Laboratory, Wellcome Trust Centre for Human Genetics, University of OxfordUK; 2 Department of Surgery, Croydon University HospitalUK; 3 Department of Cancer Genetics, St George's University of LondonUK; 4 Department of Histopathology, Hammersmith Hospital, Imperial College Healthcare NHS TrustLondon, UK; 5 NIHR Comprehensive Biomedical Research Centre, Wellcome Trust Centre for Human Genetics, University of OxfordUK

**Keywords:** whole-genome methylation, colorectal cancer

## Abstract

Changes in DNA methylation, whether hypo- or hypermethylation, have been shown to be associated with the progression of colorectal cancer. Methylation changes substantially in the progression from normal mucosa to adenoma and to carcinoma. This phenomenon has not been studied extensively and studies have been restricted to individual CpG islands, rather than taking a whole-genome approach. We aimed to study genome-wide methylation changes in colorectal cancer. We obtained 10 fresh-frozen normal tissue–cancer sample pairs, and five fresh-frozen adenoma samples. These were run on the lllumina HumanMethylation27 whole-genome methylation analysis system. Differential methylation between normal tissue, adenoma and carcinoma was analysed using Bayesian regression modelling, gene set enrichment analysis (GSEA) and hierarchical clustering (HC). The highest-rated individual gene for differential methylation in carcinomas versus normal tissue and adenomas versus normal tissue was *GRASP* (*p*_adjusted_ = 1.59 × 10^–5^, BF = 12.62, *p*_adjusted_ = 1.68 × 10^–6^, BF = 14.53). The highest-rated gene when comparing carcinomas versus adenomas was *ATM* (*p*_adjusted_ = 2.0 × 10^–4^, BF = 10.17). Hierarchical clustering demonstrated poor clustering by the CIMP criteria for methylation. GSEA demonstrated methylation changes in the Netrin–DCC and SLIT–ROBO pathways. Widespread changes in DNA methylation are seen in the transition from adenoma to carcinoma. The finding that *GRASP*, which encodes the general receptor for phosphoinositide 1-associated scaffold protein, was differentially methylated in colorectal cancer is interesting. This may be a potential biomarker for colorectal cancer. Copyright © 2012 Pathological Society of Great Britain and Ireland. Published by John Wiley & Sons, Ltd.

## Introduction

Epigenetic modification of DNA has been increasingly recognized as performing an important role in carcinogenesis. However, there are surprisingly few studies of genome-wide methylation and its effects in colorectal cancer (CRC). Initial studies of gene-specific promoter methylation 1 described the phenomenon of the CpG island phenotype (CIMP), in which multiple tumour suppressor genes, such as *CDKN2A* and *MLH1*, were aberrantly methylated at their promoter regions. CIMP tumours are known to have distinct clinical and pathological characteristics. These include 2 high rates of microsatellite instability (MSI) and *BRAF* mutations, as well as being predominantly in the right side of the colon.

Extensive work has been carried out to investigate the characteristics of CIMP tumours. Ogino *et al* 2 examined DNA methylation in five gene promoter regions (*CACNA1G*, *CDKN2A*, *CRABP1*, *MLH1* and *NEUROG1*) in a large set of 840 population-based colorectal cancer samples. They defined several types of CIMP, including CIMP-low (1–3/5 promoter regions methylated). CIMP-low tumours had significantly more *KRAS* mutations as compared to other groups, CIMP-high (≥ 4/5 promoter regions methylated) or CIMP-negative (0/5 promoter regions methylated).

Nosho *et al* 3 verified these categories, also validating the finding that CIMP-high tumours were predominantly poorly differentiated, proximally located, microsatellite unstable and frequently had *BRAF* mutations. In examining CIMP-negative tumours, Ogino *et al* 4 and Goel *et al* 5 found that tumours belonging to this group frequently demonstrated loss of heterozygosity at 18q, indicating chromosomal instability (CIN). This phenomenon and the associations observed with CIMP-low and CIMP-high tumours have been reproduced independently by other groups 6–9.

Yagi *et al* 10 carried out methylated DNA immunoprecipitation-on-chip (MeDIP-Chip) analysis of HCT116 and SW480 CRC cell lines and coupled this with whole-genome expression array analysis in a set of primary CRCs. Although they demonstrated some associations between low levels of CIMP and *KRAS* mutation, their study had a very high level of bias, due to the use of only two cell lines, with potential bias due to methylation differences in cell lines.

Ang *et al* 11 utilized an Illumina Goldengate methylation array to examine the methylation status of 1505 CpG islands. Examining fresh-frozen tissue from 91 CRC samples against 28 randomly selected, normal colorectal tissue samples, they found that 202 CpG islands were differentially methylated in tumours. Their top four differentially methylated genes – comparing methylation between tumour and normal mucosa – were *EYA4*, *HS3ST2*, *TFPI2* and *SLIT2*. Using unsupervized hierarchical clustering analysis, they found that differential methylation segregated into CIMP-H, -M and –L; however, their findings have not been independently reproduced and their study may have experienced bias due to small sample size.

Hinoue *et al* 12 analysed 120 tumour samples, a minority of which were paired with corresponding normal tissues, utilizing the Illumina HumanMethylation27 platform. They described three biologically distinct groups. The first, CIMP-H, showed a strong association with hypermethylation of *MLH1* and the *BRAF* V600E mutation. The second, CIMP-L, was enriched for *KRAS* mutations. In the third group, two distinct subgroups were found, the first with a high frequency of *TP53* mutations and association with the distal colon, and the second with low-level DNA hypermethylation and an association with the rectum. This study was primarily geared towards analysis of patterns of gene expression in relation to CpG island hypermethylation. A paired analysis of methylation was not carried out, and no premalignant lesions were analysed.

Yamauchi *et al* 13 recently carried out a study which examined the rates of CIMP, *KRAS* and *BRAF* mutations and microsatellite instabilities (MSIs) compared to the location of tumours in the colon or rectum. They found that rates of CIMP, MSI and *BRAF* mutation increased from the distal rectum towards the proximal colon. They suggested that these changes demonstrated that methylation occurs as a continuum in the colon, rather than a simple proximal/distal tumour divide, as previously described. They also found that cecal tumours represented a unique subtype of colorectal cancer, possessing high *KRAS* mutation frequency but lower CIMP-high frequency than in ascending colon tumours.

A further study by the Cancer Genome Atlas Network 14 examined methylation patterns in 276 colorectal tumours as part of a larger study. Using unsupervised hierarchical clustering, it found that tumours segregated into four groups. The first two, CIMP-high and CIMP-low, were identical to those previously described. It additionally described two other groups, which consisted of tumours that were not hypermethylated but had a higher frequency of *APC* and *TP53* mutations than the CIMP-high and -low groups.

We aimed to carry out a whole-genome methylation analysis of paired CRC and normal tissue, as well as colorectal adenomas to identify changes in patterns of methylation in each of these lesions.

## Materials and methods

### Patient recruitment

Adenomas were sampled at Hammersmith Hospital from patients undergoing colonoscopy. CRCs and paired normal tissues were from patients at St George's Hospital undergoing a resection of their tumours. The clinical and pathological characteristics of the cancers and adenomas are shown in Tables 1 and 2. At the time of surgery, resection specimens were immediately placed on ice and conveyed to a specialist histopathologist, where the specimen was opened and a representative sample of adenoma/cancer taken. For the cancers, paired normal colonic mucosa was taken as far as possible from the site of the tumour. Prior to freezing, confirmatory H&E-stained frozen sections were taken to confirm that tumour or normal tissue was present. The specimens were immediately snap-frozen in liquid nitrogen and then stored at −80 °C until use. Ethical approval was obtained from Oxfordshire Research Ethics Committee (MREC 05/Q1605/66).

**Table 1:** Details of carcinomas

**Tumour ID**	**Age**	**TNM stage**	**Duke stage**	**Site**
20 T	85	T4N0M0	B	Caecum
21 T	77	T2N2M1	D	Rectum
22 T	85	T3N0M0	B2	Caecum
23 T	94	T2N0M0	B1	Rectum
24 T	84	T3N2MX	C2	Sigmoid
26 T	51	T4N2MX	C2	Rectum
27 T	66	T3N1M0	C2	Sigmoid
28 T	80	T4N0M0	B	Descending colon
29 T	58	T3N0M0	B2	Sigmoid
30 T	48	T3N0M0	B2	Caecum

**Table 2:** Details of adenomas

**ID**	**Age**	**Dysplasia**	**Location**	**Morphology**
Ad1	74	Severe	Rectum	Tubulovillous
Ad2	64	Mild	Colon	Tubulovillous
Ad3	84	Moderate	Colon	Tubulovillous
Ad4	84	Moderate	Colon	Tubulovillous
Ad5	54	Moderate	Colon	Tubulovillous

### DNA extraction and processing

DNA was extracted using proteinase K digestion at 55 °C over 2 days in 180 µl buffer ATL and was purified using a Qiagen DNEasy kit. DNA samples were quantified using spectrophotometry. DNA samples were diluted to 50 ng/µl, using nuclease-free water, and stored at −20 °C until use. Bisulphite conversion of DNA was carried out a EZ Methylation Kit (Zymo Labs) according to the manufacturer's protocol. Quantitation of converted bisulphite DNA and assessment of completeness of conversion were carried out using qRT–PCR, according to the method of Campan *et al* 15, using primers specific for ALU repeats and for 0% and 100% bisulphite conversion, against a 100% methylated DNA and 100% unmethylated DNA reference.

### Illumina methylation array analysis

Whole-genome methylation analysis was carried out using the Illumina HumanMethylation27 array system. 1.1 µg tumour/adenoma/normal DNA was bisulphite-converted according to the recommended protocol, quantified and quality-checked as above. The converted DNA was whole-genome amplified and hybridized to the array, which was processed according to the manufacturer's protocol. The arrays were then scanned using the Illumina BeadArray system. Standard array quality checks were performed post-scanning, using Illumina GenomeStudio software, and only samples that passed internal QC were taken forward for analysis. Raw probe data were exported from GenomeStudio into R 2.9.0. The percentage methylation *β* (*β*, %methylation) at each CpG island was calculated using the internal proprietary algorithm and normalization carried out initially using the IlluminaCustom model, and with samples being divided into two groups, tumours versus normals and adenomas versus normals. Probes that failed quality control (detection *p* value > 0.05) were removed, as well as all X chromosome probes, due to hemi-methylation of these probes in female patients.

Data were exported from GenomeStudio into R and the data normalized successfully, using the *normalizeMethyLumiSet* function of *methylumiR*. A least-squares linear fit model was performed on the methylation dataset for each group. The model estimates were then transformed into moderated *t*-statistics and log-odds of differential expression by empirical Bayes shrinkage of the standard errors towards a common value. For cancers and normals this was carried out as a paired analysis, but for adenomas this analysis was carried out in an unpaired fashion, as no paired samples existed for adenomas and normals, reducing the power of the analysis. The top 100 probes based on the highest BF were identified and QQ and volcano plots produced for the entire probe set.

Hierarchical clustering (HC) of the datasets was performed using the clustering function of MultiExperiment Viewer (TMEV), part of the TM4 Microarray Software Suite 16. GSEA was carried out using GSEA v 2.1 17 (Broad Institute, MIT, USA).

### *KRAS*, *BRAF* and MSI status

Mutation analysis of codons 12,13 and 146 of *KRAS* and codon 600 of *BRAF* was carried out by direct sequencing (details available on request). For microsatellite instability (MSI) analysis, only the BAT25 and BAT26 alleles were studied. Samples were said to be MSI if they possessed one or more additional alleles at either locus compared with a control microsatellite-stable DNA. Results for *KRAS*, *BRAF* and MSI status are shown in Table 3.

**Table 3:** Mutation status of tumours analysed

**Sample**	**MSI**	***KRAS*** **codon 12**	***KRAS*** **codon 13**	***KRAS*** **codon 146**	***BRAF*** **V600E**
20 T	MSS	c.35 G > A (p.G12D)	WT	WT	WT
21 T	MSS	WT	WT	WT	WT
22 T	MSS	c.35 G > A (p.G12D)	WT	WT	WT
23 T	MSS	WT	WT	WT	WT
24 T	MSS	WT	WT	WT	WT
26 T	MSS	WT	WT	WT	WT
27 T	MSS	WT	WT	WT	WT
28 T	MSS	WT	WT	WT	WT
29 T	MSI	WT	WT	WT	c.1799 T > A (p.V600E)
30 T	MSS	WT	c.38 G > A (p. G13D)	WT	WT
Ad1	MSS	WT	WT	WT	WT
Ad2	MSS	WT	WT	WT	WT
Ad3	MSS	WT	WT	WT	WT
Ad4	MSI	c.35 G > A (p.G12D)	WT	WT	V600E
Ad5	MSS	WT	WT	WT	WT

### Correlation between promoter methylation and expression of genes

In order to ascertain whether observed differential methylation changes correlated with changes in expression of the gene, we used publically available datasets for methylation and expression from Hinoue *et al* 12. This dataset used the Illumina HumanMethylation27 array to measure whole-genome methylation (GEO Accession No. GSE25062) and the Illumina Ref-8 whole-genome expression array to measure expression (GEO Accession No. GSE25070). Expression and methylation values were correlated using linear regression.

## Results

**Figure 1:**
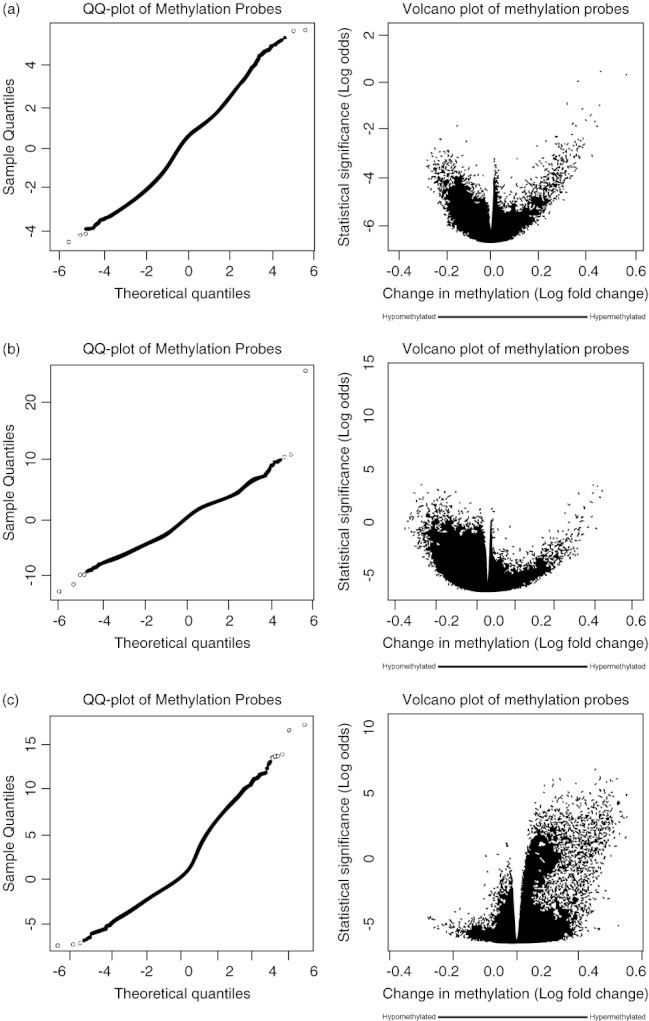
QQ and volcano plots of Bayesian analyses of differential methylation for cancers versus normal samples (a), adenomas versus normal samples (b) and cancers versus adenomas (c). The QQ plots (left) demonstrate probes that are hypermethylated (above the black normal distribution line on the left of the plot) and hypomethylated (below the normal line on the left of the plot). The volcano plot (right) demonstrates that both significantly hypomethylated and hypermethylated probes exist when comparing cancers with normal tissues, The top five probe identifiers are shown in blue text on the volcano plots, and that the five most significantly differentially methylated probes all become hypermethylated.

When comparing carcinomas and paired normal tissue, we found that > 2000 genes were differentially methylated at *p*_corrected_ < 0.05 (Figure 1a). Overall results are shown in Tables S1–S3 (see Supplementary material). On examining the volcano plots of each cohort, a number of interesting features were found. Comparing adenomas with normal tissue, the majority of genes did not change significantly or became hypomethylated in the transition from normal tissue to adenoma. In contrast, however, comparing normal tissue with carcinoma, there was a shift towards increased methylation in promoter regions in the latter, an observation confirmed by the larger proportion of genes that were methylated in carcinomas versus adenomas. These results suggest that the bulk of promoter methylation occurs around the time of transition from colorectal adenoma to carcinoma.

The highest-rated individual gene for differential methylation in carcinomas versus normals was *GRASP* (*p*_adjusted_ = 1.59 × 10^–5^, BF = 12.62), which encodes the general receptor for phosphoinositides-1-associated scaffold protein. *GRASP* was also the highest-rated gene when comparing adenomas and normal tissue (*p*_adjusted_ = 1.68 × 10^–6^, BF = 14.53). The highest-rated gene when comparing carcinomas versus adenomas was *ATM* (*p*_adjusted_ = 2.0 × 10^–4^, BF = 10.17). Volcano and QQ plots for each three groups of methylation comparisons are shown in Figure 1a–c.

Comparison of methylation and expression for *GRASP* was carried out using the dataset of Hinoue *et al* 12. This demonstrated a strong negative correlation (*coef*_logexpr_^GRASP^ = −2.95, *t* = −2.66, *p* = 0.01), ie promoter methylation of *GRASP* led to reduced expression. We found no correlation between ATM promoter methylation and expression, in common with many other studies showing that ATM expression may not be controlled by methylation in the ATM promoter 18–20.

### Hierarchical clustering (HC) into CIMP groups

We studied the effectiveness of commonly-used CIMP gene sets in ascertaining whether CIMP was a true reflection of whole-genome methylation and whether methylation patterns in our study segregated into CIMP, as previously observed. CIMP genes were defined, as those given in the studies of Toyota 1, Yagi 10 and Weisenberger 21, as *CACNA1G*, *CDKN2A*, *IGF2*, *MLH1*, *NEUROG1*, *RUNX3* and *SOCS1*. Because of their position outside CpG islands and lack of coverage on the HumanMethylation27 array, *MINT-1*, *MINT-2* and *MINT-31* were not included in the analysis. A median methylation value (*β*_median_) was calculated for all the normal mucosa samples in the dataset, and each *β*_tumour_ value was subtracted from this. Hierarchical clustering was then performed on the samples, using a Pearson correlation metric and average linkage clustering to generate a heat map and clusters for the samples.

**Figure 2:**
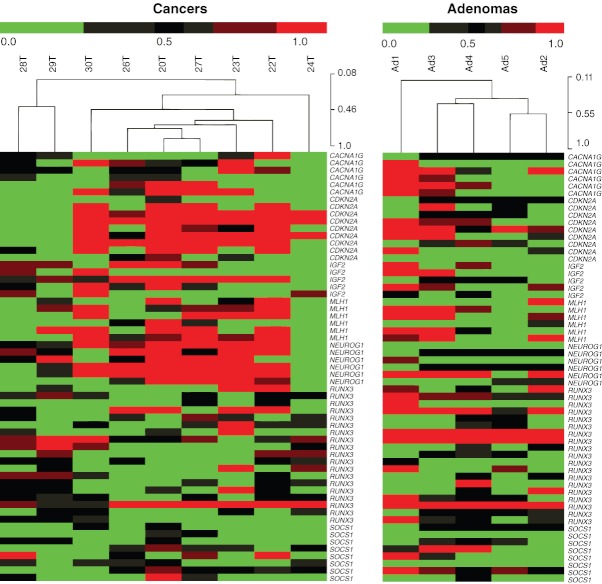
Hierarchical clustering diagram of CIMP probes for cancers and adenomas. In cancers (left) there is clustering into two groups, which correspond to the CIMP-intermediate and CIMP-low groups previously described by Yagi *et al* 10. In the adenomas (right) there is clustering into two groups, with a single sample (Ad1) having extensive methylation compatible with CIMP and another group demonstrating low levels of methylation: red, hypermethylation; green, hypomethylation; black, no change.

The heat map for clustering by CIMP probes is shown in Figure 2 for carcinomas and adenomas. In the cancer group, there appeared to be three distinct clusters, with a low methylation cluster (cancers 28 T and 29 T), an intermediate methylation cluster (24 T) and a high methylation cluster (20 T, 22 T, 23 T, 26 T, 27 T and 30 T).

For the cancer samples, we initially compared our clusters with the conventional definition of CIMP of Weisenberger *et al* 21 and Toyota *et al* 1, viz. that ≥ 3/5 CIMP-related genes should be methylated to call a sample CIMP-H, a trait that is usually associated with *BRAF* mutation. In our group of tumours, no sample fitted this definition of CIMP-H using *ab initio* hierarchical clustering. We observed that the CIMP-intermediate phenotype observed by Yagi *et al* 10 would correspond with the group of tumours that clustered into a high methylation group seen in our series. Within these cancers, 20 T and 22 T possessed *KRAS* mutations, an association also observed by Yagi in the CIMP-intermediate group. We also found that in tumour 29 T, although it was *BRAF* mutant and microsatellite unstable, there were low levels of methylation, in contrast to other studies.

In the adenoma dataset, the pattern of hierarchical clustering was clearer, with segregation into two main clusters, one with high levels of methylation, which fitted with the definition of CIMP-H by Weisenberger and Toyota. There was one other cluster with lower levels of methylation, conforming to the CIMP-L epigenotype. In this dataset, the MSI^+^, *BRAF*-mutant adenoma (Ad4) was not associated with high levels of methylation (Ad1).

### Gene set enrichment analysis (GSEA)

GSEA to determine sets of genes that tended to be hypo/hypermethylated was performed for carcinomas versus normal tissue and adenomas versus normal tissue. The C5 GO gene set was chosen for the analysis, which consists of 1454 curated gene sets divided by molecular function. As more than one probe was present for each CpG island, and because there may have been multiple probes for each gene, probes for the same gene were collapsed in the analysis, and 1000 GSEA iterations were carried out.

### GSEA comparing carcinomas versus normal tissue

In total, 569/938 gene sets were hypermethylated and 369 gene sets were hypomethylated. The top 10 hypermethylated sets (by normalized enrichment score, NES) are shown in Table S4 (see Supplementary material). The overall false-discovery rate (FDR) statistic was 0.31, missing the threshold for significance set by Subramanian *et al* 17 of FDR < 0.25. One gene set was significantly enriched at *p <* 0.01 (FDR = 0.24) and 11 gene sets were significantly enriched at *p <* 0.05 (FDR = 0.31). The top-ranked gene set was the extracellular matrix structural constituent gene set and the top-ranked gene within this was *FBN2* (Fibrillin-2), which has been proposed previously as a marker for tumour methylation by Yagi *et al* 10. Within the third-ranked gene set, the *GRM* series of metabotropic glutamate receptors was highly ranked, a set of genes which has been identified as being associated with altered responses to 5-FU chemotherapy 22.

A leading-edge analysis was then carried out in GSEA (leading-edge plots in Figures S1 and S2; see Supplementary material) and the top 20 datasets ranked by NES were chosen. The top-ranked gene in the leading-edge analysis was *SLIT2* (slit homologue 2), appearing in six gene sets. *SLIT2* interacts with netrin-1 (also known as Deleted in Colorectal Cancer), a possible tumour suppressor 23 that has previously been identified in other methylation studies of CRC 11. Other genes of interest in CRC seen in the leading-edge analysis included *SLIT1*, *TGFBR2*, *PAX2*, *UNC5C*, *OTX2*, *NGNT1* and *GDNF1*.

### GSEA comparing adenomas and normal tissue

GSEA was then carried out comparing adenomas with normal mucosa. 118/938 gene sets were hypermethylated and 118/938 became hypermethylated, with no gene sets meeting the FDR < 25% threshold. However, 21 hypomethylated gene sets were significantly enriched at *p <* 0.01 and 98 were enriched at *p* < 0.05. The top 20 sets are shown in Table S5 (see Supplementary material). In these sets, the top-ranked set was Viral Genome Replication, a set that includes *UBP1* and *EIF5A* (eukaryotic translation initiation factor 5A).

The 98 significantly enriched gene sets were then taken forward into a leading-edge analysis to identify common genes that were hypomethylated. The genes of interest within this subset included the top-rated *NDUFA13* (NADH dehydrogenase ubiquinone 1*α* subcomplex subunit 13), represented in 20 gene sets. This gene has been shown to interact with *STAT5* 24 and therefore the *JAK–STAT–EGFR* pathway, which has been associated with a worse prognosis in CRC 25.

## Discussion

We have carried out whole-genome methylation analysis of paired colorectal tumour and normal tissues, and have undertaken a comparison with unpaired colorectal adenomas. A strength of our study was the use of paired tumour–normal mucosa as well as premalignant adenomas. Our study suffers from small sample numbers, due to the difficulties of obtaining this type of material, and therefore introduces potential bias. Our study also focused solely on human tumour material, and did not take account of the tumour microenvironment (ie tumour-associated fibroblasts), as described by Allen *et al* 26. This microenvironment could have modulatory effects on tumour behaviour in addition to promoter methylation and could also contaminate any methylation analysis.

Using Bayesian analysis, we found a strong evidence of *GRASP* promoter methylation in carcinomas and adenomas. We have also shown that *GRASP* promoter methylation is significantly correlated with GRASP expression. As we found that GRASP becomes methylated in both adenomas and carcinomas, it is reasonable to assume that its promoter methylation occurs at an early stage in the adenoma–carcinoma sequence. *GRASP* is located at chromosome 12q13.13 and encodes a 395 amono acid protein involved in p14ARF signalling 27. The normal function of *GRASP* is to promote *ARF*–Rac signalling. p14ARF, which is encoded by the *CDKN2A* (*INK4a*) tumour suppressor gene, acts as a checkpoint within the ARF–MDM2–p53 pathway 28, in that normal expression of p14ARF is required to activate and stabilize p53, which can interrupt the cell cycle if needed when a mutagenic event occurs 29, as shown in Figure S3 (see Supplementary material). We also found that the promoter region of *ATM* is significantly methylated in the transition from adenoma to carcinoma; however, this does not correlate with expression of ATM. Not all gene promoter regions control expression of that gene and it is possible that control of expression of ATM is via another mechanism, given the heterogeneity of data 18–20 comparing *ATM* promoter methylation and expression.

We found that the bulk of promoter methylation occurs in the transition from adenoma to carcinoma, rather than in that from normal tissue to adenoma. Moreover, hypomethylation occurs in the transition from normal tissue to adenoma, whereas hypermethylation is mostly associated with the transition from adenoma to carcinoma. A few other studies have examined the temporal relationship of promoter methylation to colorectal carcinogenesis, but these have mainly examined CIMP genes. For example, Ahlquist *et al* 30 examined the methylation of a set of 11 genes made up of a mixture of CIMP genes and others identified as significant in methylation studies. They found that the average number of methylated genes was 0.4 in normal mucosa, but 2.2 in adenomas and 3.9 in carcinomas. They suggested that the bulk of methylation occurs during adenoma formation. However, our cluster data suggest that CIMP is not fully representative of whole-genome methylation. None of the cancers in this study reached the conventional threshold for CIMP positivity 21, although we did observe the reported association between *KRAS* mutation and a 'CIMP-low' phenotype 10. We also agreed with the findings of Yamauchi *et al* 13, as all our cecal tumours possessed *KRAS* mutations, with variable levels of methylation.

One of the main roles of GSEA is that it can highlight groups of biological pathways for further investigation. For example, in the GSEA for the normal–carcinoma group, cellular pathways concerned with signalling (cAMP, tyrosine kinase and G-protein-coupled receptors) were significantly enriched in the tumours. The metabotropic glutamate receptors (GRM) were significantly over-represented in the GSEA analysis, and these have been implicated in chemotherapy resistance in a number of tumours, including CRC 22, melanoma 31 and glioblastoma 32. The leading-edge analysis of the CRC methylation dataset revealed that extensive methylation occurs in the DCC–netrin pathway. *SLIT2* (SLIT2 homologue 2 protein), as well as *UNC5C*, are methylated in the CRC samples in this study. *SLIT2* has been shown to be a negative regulatory component of the *DCC*–netrin pathway 33, and normal expression of *SLIT2* has been shown to repress growth and metastasis of squamous cell carcinomas and fibrosarcomas 23.

In conclusion, the use of whole-genome methylation analysis has highlighted a number of genes and pathways, some of which have not been suggested to have a role in CRC. *GRASP* is one such interesting candidate. We have also demonstrated that promoter methylation occurs mainly in the transition from adenoma to carcinoma, suggesting that epigenetic events may be more important in promoting than initiating colorectal tumorigenesis.

## SUPPORTING INFORMATION ON THE INTERNET

The following supporting information may be found in the online version of this article.

**Figure S1.** Leading edge diagram for carcinoma versus normal tissue, demonstrating the number of times a gene has a significant change in methylation in each set. *SLIT2*, the highest-ranked gene, is noted to be significantly hypermethylated in six different gene sets.

**Figure S2.** Leading edge diagram of adenoma versus normal tissue, demonstrating the number of times a gene is significantly hypomethylated within a gene set. The top-ranked gene, *NDUFA13*, occurs in 20 different gene sets.

**Figure S3.** Representation of GRASP/CDKN2A (p14ARF)/TP53 pathway.

**Table S1.** Top 25 differentially methylated genes from Bayesian model of carcinomas versus normal tissue.

**Table S2.** Top 25 differentially methylated genes from Bayesian model of adenomas versus normal tissue.

**Table S3.** Top 25 differentially methylated genes from Bayesian model of carcinomas versus adenomas.

**Table S4.** Table of gene set ranked by NES score for cancers versus normals in methylated probes.

**Table S5.** Table of gene sets in adenomas ranked by NES score for adenomas versus normals in demethylated probes.
