# Supplementary figures and images for: Whole-genome methylation analysis of benign and malignant colorectal tumours
Source: J Pathol. 2013 Jan 24;229(5):697–704. doi: 10.1002/path.4132 (PMC3619233; doi:10.1002/path.4132)

Figure S1


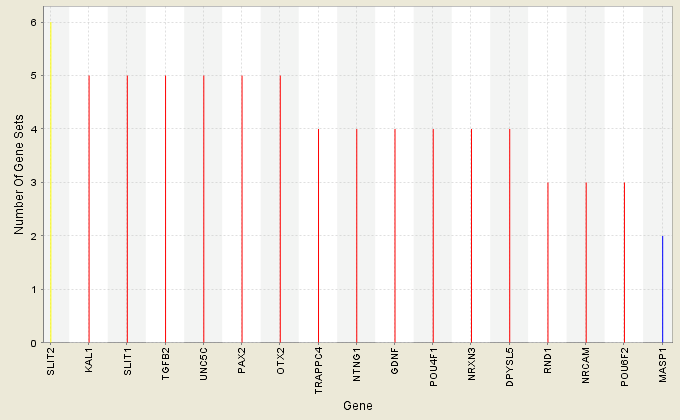

Supplement: Supplementary file 1 [file path0229-0697-SD1.doc]
