# Supplementary material for: Whole-genome methylation analysis of benign and malignant colorectal tumours
Source: J Pathol. 2013 Jan 24;229(5):697–704. doi: 10.1002/path.4132 (PMC3619233; doi:10.1002/path.4132)
Supplement: Supplementary file 4 [file path0229-0697-SD4.doc]

**Table S1.** Top 25 differentially methylated genes from Bayesian model of carcinomas versus normal tissue

| **Rank** | **Gene** | **Ave meth** | ***p*** | **adj. *p*** | **BF** | **Function** | **Chr** |
| --- | --- | --- | --- | --- | --- | --- | --- |
| 1 | *GRASP* | 0.47 | 6.01 × 10^–10^ | 1.59 × 10^–5^ | 12.62 | GRP1 (general receptor for phosphoinositides 1)-associated scaffold protein | 12 |
| 2 | *C1orf165* | 0.40 | 1.04 × 10^–7^ | 0.001 | 8.28 | hypothetical protein LOC79656 | 1 |
| 3 | *GFRA1* | 0.36 | 1.60 × 10^–7^ | 0.001 | 7.89 | GDNF family receptor *α*1 isoform a preproprotein | 10 |
| 4 | *SLC35F3* | 0.51 | 2.27 × 10^–7^ | 0.001 | 7.56 | solute carrier family 35; member F3 | 1 |
| 5 | *ELOVL2* | 0.40 | 3.34 × 10^–7^ | 0.001 | 7.20 | elongation of very long chain fatty acids (FEN1/Elo2; SUR4/Elo3; yeast)-like 2 | 6 |
| 6 | *HSPA1A* | 0.43 | 3.87 × 10^–7^ | 0.001 | 7.06 | heat shock 70 kDa protein 1A | 6 |
| 7 | *VSX1* | 0.40 | 4.03 × 10^–7^ | 0.001 | 7.02 | visual system homeobox 1 protein isoform a | 20 |
| 8 | *SNAP91* | 0.39 | 4.25 × 10^–7^ | 0.001 | 6.97 | synaptosomal-associated protein; 91 kDa homologue | 6 |
| 9 | *SLC4A11* | 0.58 | 5.19 × 10^–7^ | 0.002 | 6.78 | solute carrier family 4 member 11 | 20 |
| 10 | *BTG4* | 0.50 | 6.37 × 10^–7^ | 0.002 | 6.58 | B cell translocation gene 4 | 11 |
| 11 | *PDE8B* | 0.39 | 7.76 × 10^–7^ | 0.002 | 6.39 | phosphodiesterase 8B isoform 1 | 5 |
| 12 | *FOXE1* | 0.41 | 8.88 × 10^–7^ | 0.002 | 6.26 | forkhead box E1 | 9 |
| 13 | *CD8A* | 0.40 | 9.67 × 10^–7^ | 0.002 | 6.18 | CD8 antigen *α* polypeptide isoform 1 precursor | 2 |
| 14 | *EYA4* | 0.37 | 1.50 × 10^–7^ | 0.003 | 5.76 | eyes absent 4 isoform a | 6 |
| 15 | *EYA4* | 0.34 | 1.97 × 10^–7^ | 0.003 | 5.49 | eyes absent 4 isoform a | 6 |
| 16 | *SLC16A12* | 0.38 | 2.63 × 10^–7^ | 0.004 | 5.21 | solute carrier family 16 (monocarboxylic acid transporters); member 12 | 10 |
| 17 | *HS3ST3A1* | 0.45 | 2.81 × 10^–6^ | 0.004 | 5.14 | heparansulphate d-glucosaminyl 3-*O*-sulphotransferase 3A1 | 17 |
| 18 | *ZNF625* | 0.49 | 2.90 × 10^–6^ | 0.004 | 5.11 | zinc finger protein 625 | 19 |
| 19 | *PTPRM* | 0.40 | 2.99 × 10^–6^ | 0.004 | 5.08 | protein tyrosine phosphatase; receptor type; M precursor | 18 |
| 20 | *IRF4* | 0.38 | 3.21 × 10^–6^ | 0.004 | 5.01 | interferon regulatory factor 4 | 6 |
| 21 | *C20orf39* | 0.35 | 3.48 × 10^–6^ | 0.004 | 4.93 | hypothetical protein LOC79953 | 20 |
| 22 | *T* | 0.36 | 3.86 × 10^–6^ | 0.005 | 4.83 | transcription factor T | 6 |
| 23 | *TIAM1* | 0.33 | 4.11 × 10^–6^ | 0.005 | 4.77 | T cell lymphoma invasion and metastasis 1 | 21 |
| 24 | *VGCNL1* | 0.56 | 4.16 × 10^–6^ | 0.005 | 4.76 | voltage gated channel-like 1 | 13 |
| 25 | *MDFI* | 0.43 | 4.45 × 10^–6^ | 0.005 | 4.69 | MyoD family inhibitor | 6 |
