# Supplementary material for: Whole-genome methylation analysis of benign and malignant colorectal tumours
Source: J Pathol. 2013 Jan 24;229(5):697–704. doi: 10.1002/path.4132 (PMC3619233; doi:10.1002/path.4132)
Supplement: Supplementary file 5 [file path0229-0697-SD5.doc]

**Table S2.** Top 25 differentially methylated genes from Bayesian model of adenomas versus normal tissue

| **Rank** | **Gene** | ***Δ* Meth** | ***p*** | **adj. *p*** | **BF** | **Function** | **Chr** |
| --- | --- | --- | --- | --- | --- | --- | --- |
| 1 | *GRASP* | 0.42 | 6.11 × 10^–11^ | 1.68 × 10^–6^ | 14.53 | GRP1 (general receptor for phosphoinositides 1)-associated scaffold protein | 12 |
| 2 | *C3AR1* | 0.60 | 1.09 × 10^–7^ | 0.002 | 8.31 | complement component 3a receptor 1 | 12 |
| 3 | *KRTAP17-1* | 0.31 | 3.07 × 10^–7^ | 0.002 | 7.32 | keratin associated protein 17-1 | 17 |
| 4 | *ADHFE1* | 0.38 | 3.49 × 10^–7^ | 0.002 | 7.19 | alcohol dehydrogenase; iron containing; 1 | 8 |
| 5 | *IRX2* | 0.35 | 5.10 × 10^–7^ | 0.003 | 6.83 | iroquoishomeobox protein 2 | 5 |
| 6 | *VSX1* | 0.37 | 8.23 × 10^–7^ | 0.004 | 6.36 | visual system homeobox 1 protein isoform a | 20 |
| 7 | *BTG4* | 0.45 | 1.13 × 10^–6^ | 0.004 | 6.04 | B cell translocation gene 4 | 11 |
| 8 | *ZNF625* | 0.44 | 1.14 × 10^–6^ | 0.004 | 6.03 | zinc finger protein 625 | 19 |
| 9 | *ANKRD13C* | 0.56 | 1.48 × 10^–6^ | 0.004 | 5.78 | ankyrin repeat domain 13C | 1 |
| 10 | *PDILT* | 0.88 | 1.49 × 10^–6^ | 0.004 | 5.77 | protein disulphideisomerase-like protein of the testis | 16 |
| 11 | *NPR3* | 0.35 | 2.00 × 10^–6^ | 0.005 | 5.48 | natriuretic peptide receptor C/guanylatecyclase C (atrionatriuretic peptide receptor C) | 5 |
| 12 | *SFRP2* | 0.36 | 2.14 × 10^–6^ | 0.005 | 5.41 | Secreted frizzled-related protein 2 | 4 |
| 13 | *VPREB1* | 0.31 | 2.68 × 10^–6^ | 0.006 | 5.19 | immunoglobulin iota chain preproprotein | 22 |
| 14 | *ERAF* | 0.63 | 3.44 × 10^–6^ | 0.006 | 4.94 | erythroid associated factor | 16 |
| 15 | *KCNN4* | 0.62 | 3.54 × 10^–6^ | 0.006 | 4.91 | intermediate conductance calcium-activated potassium channel protein 1 | 19 |
| 16 | *DAB2IP* | 0.28 | 3.73 × 10^–6^ | 0.006 | 4.86 | Disabled homologue 2-interacting protein | 9 |
| 17 | *GPX5* | 0.36 | 5.44 × 10^–6^ | 0.008 | 4.48 | glutathione peroxidase 5 precursor; isoform 1 | 6 |
| 18 | *GLMN* | 0.36 | 5.47 × 10^–6^ | 0.008 | 4.47 | glomulin isoform FAP68 | 1 |
| 19 | *OR10A5* | 0.75 | 5.99 × 10^–6^ | 0.009 | 4.38 | olfactory receptor; family 10; subfamily A; member 5 | 11 |
| 20 | *FZD2* | 0.35 | 6.85 × 10^–6^ | 0.009 | 4.25 | frizzled 2 | 17 |
| 21 | *DFNA5* | 0.28 | 7.08 × 10^–6^ | 0.009 | 4.21 | deafness; autosomal dominant 5 protein | 7 |
| 22 | *MLCK* | 0.14 | 7.22 × 10^–6^ | 0.009 | 4.19 | MLCK protein | 16 |
| 23 | *TLX3* | 0.46 | 9.78 × 10^–6^ | 0.011 | 3.89 | T cell leukaemia; homeobox 3 | 5 |
| 24 | *ZNF222* | 0.59 | 1.09 × 10^–5^ | 0.011 | 3.77 | zinc finger protein 222 | 19 |
| 25 | *HHLA1* | 0.85 | 1.10 × 10^–5^ | 0.011 | 3.76 | HERV-H LTR-associating 1 | 8 |
