# Supplementary material for: Whole-genome methylation analysis of benign and malignant colorectal tumours
Source: J Pathol. 2013 Jan 24;229(5):697–704. doi: 10.1002/path.4132 (PMC3619233; doi:10.1002/path.4132)
Supplement: Supplementary file 6 [file path0229-0697-SD6.doc]

**Table S3.** Top 25 differentially methylated genes from Bayesian model of carcinomas versus adenomas

| **Rank** | **Gene** | **Δ Meth** | ***p*** | **adj. *p*** | **BF** | **Product** | **Chromosome** |
| --- | --- | --- | --- | --- | --- | --- | --- |
| 1 | *ATM* | 0.23 | 1.26 × 10^–8^ | 0.0002 | 10.17 | ataxia telangiectasia mutated protein isoform 1 | 11 |
| 2 | *GLMN* | 0.37 | 1.80 × 10^–8^ | 0.0002 | 9.87 | glomulin isoform FAP68 | 1 |
| 3 | *EYA2* | 0.29 | 9.67 × 10^–8^ | 0.0006 | 8.40 | eyes absent 2 isoform a | 20 |
| 4 | *GOSR1* | 0.41 | 1.10 × 10^–7^ | 0.0006 | 8.29 | Golgi SNAP receptor complex member 1 isoform 3 | 17 |
| 5 | *C20orf20* | 0.15 | 1.12 × 10^–7^ | 0.0006 | 8.27 | MRG-binding protein | 20 |
| 6 | *NDUFA4* | 0.19 | 1.25 × 10^–7^ | 0.0006 | 8.17 | NADH dehydrogenase (ubiquinone) 1*α* subcomplex; 4; 9 kDa | 7 |
| 7 | *KIAA1219* | 0.16 | 1.73 × 10^–7^ | 0.0007 | 7.87 | hypothetical protein LOC57148 | 20 |
| 8 | *TFAP2D* | 0.42 | 2.10 × 10^–7^ | 0.0007 | 7.70 | transcription factor AP-2 *β*-like 1 | 6 |
| 9 | *C6orf89* | 0.29 | 2.92 × 10^–7^ | 0.0007 | 7.39 | hypothetical protein LOC221477 | 6 |
| 10 | *BCL2L1* | 0.31 | 4.22 × 10^–7^ | 0.0007 | 7.04 | BCL2-like 1 isoform 2 | 20 |
| 11 | *NADSYN1* | 0.53 | 4.43 × 10^–7^ | 0.0007 | 7.00 | NAD synthetase 1 | 11 |
| 12 | *C6orf151* | 0.19 | 4.55 × 10^–7^ | 0.0007 | 6.97 | U11/U12 snRNP 48K | 6 |
| 13 | *AUP1* | 0.13 | 4.69 × 10^–7^ | 0.0007 | 6.95 | ancient ubiquitous protein 1 isoform 3 | 2 |
| 14 | *MKRN1* | 0.47 | 4.80 × 10^–7^ | 0.0007 | 6.92 | makorin; ring finger protein; 1 | 7 |
| 15 | *FLJ37562* | 0.54 | 4.92 × 10^–7^ | 0.0007 | 6.90 | hypothetical protein LOC134553 | 5 |
| 16 | *ZNF12* | 0.35 | 4.94 × 10^–7^ | 0.0007 | 6.90 | zinc finger protein 12 (KOX 3) | 7 |
| 17 | *ZNF571* | 0.21 | 5.73 × 10^–7^ | 0.0007 | 6.75 | zinc finger protein 571 | 19 |
| 18 | *SPATA1* | 0.41 | 5.80 × 10^–7^ | 0.0007 | 6.74 | spermatogenesis-associated 1 | 1 |
| 19 | *ZSWIM1* | 0.21 | 5.97 × 10^–7^ | 0.0007 | 6.72 | zinc finger; SWIM domain-containing 1 | 20 |
| 20 | *DR1* | 0.30 | 6.74 × 10^–7^ | 0.0007 | 6.60 | down-regulator of transcription 1 | 1 |
| 21 | *C10orf63* | 0.19 | 7.08 × 10^–7^ | 0.0007 | 6.55 | enkurin | 10 |
| 22 | *MTRF1* | 0.35 | 7.19 × 10^–7^ | 0.0007 | 6.54 | mitochondrial translational release factor 1 | 13 |
| 23 | *GYG1* | 0.29 | 7.20 × 10^–7^ | 0.0007 | 6.54 | glycogenin | 3 |
| 24 | *CDK2* | 0.13 | 7.29 × 10^–7^ | 0.0007 | 6.53 | cyclin-dependent kinase 2 | 12 |
| 25 | *DYNC2LI1* | 0.36 | 7.30 × 10^–7^ | 0.0007 | 6.52 | dynein 2 light intermediate chain isoform 1 | 2 |
