# Supplementary material for: Whole-genome methylation analysis of benign and malignant colorectal tumours
Source: J Pathol. 2013 Jan 24;229(5):697–704. doi: 10.1002/path.4132 (PMC3619233; doi:10.1002/path.4132)
Supplement: Supplementary file 7 [file path0229-0697-SD7.doc]

**Table S4.** Gene sets ranked by NES score for cancers versus normals in methylated probes

| **Rank** | **Gene set** | **NES** | **NOM *p*** |
| --- | --- | --- | --- |
| 1 | EXTRACELLULAR_MATRIX_STRUCTURAL_CONSTITUENT | 1.7 | 0.006 |
| 2 | SYNAPTIC_TRANSMISSION | 1.59 | 0.07 |
| 3 | G_PROTEIN_SIGNALING__COUPLED_TO_CAMP_NUCLEOTIDE_SECOND_MESSENGER | 1.57 | 0.083 |
| 4 | NEURON_DIFFERENTIATION | 1.56 | 0.034 |
| 5 | REGULATION_OF_HEART_CONTRACTION | 1.54 | 0.042 |
| 6 | NEUROLOGICAL_SYSTEM_PROCESS | 1.53 | 0.06 |
| 7 | CAMP_MEDIATED_SIGNALING | 1.53 | 0.089 |
| 8 | G_PROTEIN_SIGNALING__COUPLED_TO_CYCLIC_NUCLEOTIDE_SECOND_MESSENGER | 1.52 | 0.087 |
| 9 | TRANSMEMBRANE_RECEPTOR_PROTEIN_TYROSINE_KINASE_ACTIVITY | 1.52 | 0.024 |
| 10 | CYCLIC_NUCLEOTIDE_MEDIATED_SIGNALING | 1.5 | 0.092 |
| 11 | NERVOUS_SYSTEM_DEVELOPMENT | 1.5 | 0.056 |
| 12 | GLUTAMATE_RECEPTOR_ACTIVITY | 1.5 | 0.1 |
| 13 | AMINE_RECEPTOR_ACTIVITY | 1.5 | 0.126 |
| 14 | TRANSMISSION_OF_NERVE_IMPULSE | 1.5 | 0.082 |
| 15 | GENERATION_OF_NEURONS | 1.5 | 0.034 |
| 16 | TRANSFERASE_ACTIVITY__TRANSFERRING_SULPHUR_CONTAINING_GROUPS | 1.49 | 0.061 |
| 17 | VOLTAGE_GATED_POTASSIUM_CHANNEL_COMPLEX | 1.49 | 0.08 |
| 18 | SULPHOTRANSFERASE_ACTIVITY | 1.48 | 0.071 |
| 19 | NEURON_DEVELOPMENT | 1.48 | 0.049 |
| 20 | CENTRAL_NERVOUS_SYSTEM_DEVELOPMENT | 1.47 | 0.039 |
