# Supplementary material for: Whole-genome methylation analysis of benign and malignant colorectal tumours
Source: J Pathol. 2013 Jan 24;229(5):697–704. doi: 10.1002/path.4132 (PMC3619233; doi:10.1002/path.4132)
Supplement: Supplementary file 8 [file path0229-0697-SD8.doc]

**Table S5.** Gene sets in adenomas ranked by NES score for adenomas versus normals in demethylated probes

| **Rank** | **Gene set** | **ES** | **NES** | **NOM *p*** |
| --- | --- | --- | --- | --- |
| 1 | VIRAL_GENOME_REPLICATION | –0.64 | –1.82 | 0.007 |
| 2 | VIRAL_INFECTIOUS_CYCLE | –0.6 | –1.77 | 0.002 |
| 3 | VIRAL_REPRODUCTIVE_PROCESS | –0.59 | –1.71 | 0.002 |
| 4 | N_ACYLTRANSFERASE_ACTIVITY | –0.51 | –1.69 | 0.01 |
| 5 | SPLICEOSOME_ASSEMBLY | –0.6 | –1.69 | 0.023 |
| 6 | MRNA_PROCESSING_GO_0006397 | –0.55 | –1.64 | 0.013 |
| 7 | RESPONSE_TO_BACTERIUM | –0.54 | –1.64 | 0.064 |
| 8 | N_ACETYLTRANSFERASE_ACTIVITY | –0.51 | –1.63 | 0.034 |
| 9 | GROWTH | –0.48 | –1.62 | 0.002 |
| 10 | REGULATION_OF_PROTEIN_SECRETION | –0.48 | –1.62 | 0.049 |
| 11 | DNA_DIRECTED_RNA_POLYMERASE_II__HOLOENZYME | –0.56 | –1.6 | 0 |
| 12 | DEFENSE_RESPONSE_TO_BACTERIUM | –0.53 | –1.6 | 0.045 |
| 13 | RNA_SPLICING_FACTOR_ACTIVITY__TRANSESTERIFICATION_MECHANISM | –0.66 | –1.58 | 0.037 |
| 14 | VIRAL_REPRODUCTION | –0.53 | –1.58 | 0.008 |
| 15 | TRANSLATION | –0.47 | –1.58 | 0.002 |
| 16 | POSITIVE_REGULATION_OF_TRANSCRIPTION_FROM_RNA_POLYMERASE_II_PROMOTER | –0.44 | –1.58 | 0.004 |
| 17 | TRNA_METABOLIC_PROCESS | –0.68 | –1.57 | 0 |
| 18 | REGULATION_OF_GROWTH | –0.48 | –1.57 | 0.011 |
| 19 | RNA_SPLICING__VIA_TRANSESTERIFICATION_REACTIONS | –0.53 | –1.56 | 0.041 |
| 20 | ZINC_ION_BINDING | –0.47 | –1.56 | 0 |
